# Gestodene, a novel positive allosteric modulator of PAR1, enhances PAR1-mediated human platelet aggregation

**DOI:** 10.3389/fphar.2024.1430548

**Published:** 2024-07-26

**Authors:** So-Hyeon Park, Yunkyung Heo, Il Kwon, Sungwoo Jo, Hyejin Jeon, Yechan Lee, Jieun Kim, Ji Hoe Heo, Wan Namkung

**Affiliations:** ^1^ College of Pharmacy and Yonsei Institute of Pharmaceutical Sciences, Yonsei University, Incheon, Republic of Korea; ^2^ Integrative Research Institute for Cerebrovascular and Cardiovascular Diseases, Yonsei University College of Medicine, Seoul, Republic of Korea; ^3^ Graduate Program of Industrial Pharmaceutical Science, Yonsei University, Incheon, Republic of Korea; ^4^ Department of Neurology, Yonsei University College of Medicine, Seoul, Republic of Korea

**Keywords:** PAR1, PAM, gestodene, platelet, thromboembolism

## Abstract

**Background:** Protease-activated receptor 1 (PAR1) is expressed in human platelets and can be activated by low concentrations of thrombin. Vorapaxar, a selective antagonist of PAR1, inhibits thrombin-induced calcium mobilization in human platelet, which is associated with an increased risk of bleeding. Conversely, the administration of a positive allosteric modulator (PAM) of PAR1 may pose a substantial risk of thrombosis due to inducing excessive platelet activation. In this study, we discovered a novel PAM of PAR1 and investigated the effect of enhanced PAR1 activation by PAM of PAR1 on platelet activation.

**Methods:** To find PAMs of PAR1, a cell-based screen was performed in HT29 cells, and finally, gestodene, an oral contraceptive drug (OC), was identified as a novel PAM of PAR1. The mechanism of action of gestodene and its effects on platelet activation were investigated in human megakaryocytic leukemia cell line MEG-01 cells and human platelet.

**Results:** Gestodene enhanced both thrombin- and PAR1-activating peptide (AP)-induced intracellular calcium levels in a dose-dependent manner without altering PAR2 and PAR4 activity. Gestodene significantly increased PAR1-AP-induced internalization of PAR1 and phosphorylation of ERK1/2, and the enhancing effects were significantly blocked by vorapaxar. Furthermore, gestodene potently increased PAR1-AP induced morphological changes in MEG-01 cells. Remarkably, in human blood, gestodene exerted a robust augmentation of PAR1-AP-induced platelet aggregation, and vorapaxar effectively attenuated the gestodene-induced enhancement of platelet aggregation mediated by PAR1.

**Conclusion:** Gestodene is a selective PAM of PAR1 and suggest one possible mechanism for the increased risk of venous thromboembolism associated with OCs containing gestodene.

## Introduction

Platelets are known to play an essential role in arterial thrombosis and hemostasis, and thrombin acts as the most potent platelet activator, stimulating and enhancing platelet activation and thrombosis (Angiolillo et al., 2010). Human platelets express two functional thrombin receptors, protease-activated receptor 1 (PAR1) and PAR4, which are potential targets for antiplatelet therapy (Kahn et al., 1999; Rwibasira Rudinga et al., 2018). Thrombin irreversibly activates both PAR1 and PAR4 by cleaving their N-terminal exodomains and exposing tethered ligands, and activation of either receptor is sufficient to induce platelet secretion and aggregation (Kahn et al., 1999). In contrast to PAR4, PAR1 acts as a high-affinity thrombin receptor because it contains a hirudin-like sequence that binds tightly to thrombin exosite I (Kahn et al., 1999; Bah et al., 2007). In addition, thrombin-induced activation of PAR1 results in a rapid and transient intracellular calcium response, whereas thrombin-induced activation of PAR4 results in a slow and sustained calcium mobilization (Covic et al., 2000). In addition, accumulating evidence indicates that PAR1 and PAR4 can induce platelet aggregation through distinct signaling pathways (Covic et al., 2000; Holinstat et al., 2006; Holinstat et al., 2007; Voss et al., 2007; Holinstat et al., 2009). Platelet activation by 1 nM thrombin is strongly inhibited by treatment with PAR1 antagonists, whereas platelet activation by 30 nM thrombin, a relatively high concentration, is weakly inhibited by PAR1 antagonist alone, but when PAR1 and PAR4 are simultaneously inhibited, the platelet activation by high concentrations of thrombin is significantly inhibited (Kahn et al., 1999; Judge et al., 2015). These results suggest that both PAR1 and PAR4 are promising drug targets for antiplatelet agents.

In the case of PAR1 antagonists, several potent and selective antagonists have been identified, such as vorapaxar (SCH-530348), SCH-79797, BMS-200261, and atopaxar (E5555), which exert antiplatelet effects by inhibiting the activity of PAR1 expressed on human platelets (Aisiku et al., 2015). Vorapaxar is the first PAR-1 antagonist to receive FDA approval as an oral antiplatelet agent. Although vorapaxar reduces the risk of cardiovascular death and ischemic events in patients with atherosclerosis, it also increases the risk of moderate or severe bleeding, including intracranial hemorrhage (Morrow et al., 2012). These results also suggest that drugs that induce excessive PAR1 activity in platelets have the potential to induce thrombosis. In a previous study, we showed that GB83 is the first small-molecule positive allosteric modulator (PAM) of PAR1 (Seo et al., 2021). However, GB83 is a dual acting modulator of PARs, inducing both PAR1 potentiation and PAR2 activation (Heo et al., 2022b), so it is not appropriate to see a clear effect on thrombus formation by enhancing PAR1 activity.

In a previous study, we developed an image-based assay to measure morphological changes in the human megakaryoblastic leukemia cell line MEG-01 cells and showed that MEG-01 cells express functional PAR1 and PAR4, and that activation of both PAR1 and PAR4 induces morphological changes in MEG-01 cells (Heo et al., 2022a). In this study, we identified a novel, potent and selective PAM of PAR1, gestodene, a synthetic progestogen. Interestingly, gestodene is a third-generation oral contraceptive (OC), and OC containing gestodene may increase the risk of thromboembolism (Lidegaard et al., 2011). In addition, gestodene increases PAR1 expression levels in vascular smooth muscle cells (Herkert et al., 2001). Thus, we investigated the mechanisms underlying gestodene-mediated enhancement of PAR1 activity and the effect of gestodene on human platelet aggregation.

## Materials and methods

### Materials and reagents

The compound collections used for screening included 1813 approved drugs and ticagrelor were purchased from TargetMol Chemicals Inc. (Boston, MA, United States). Gestodene, thrombin, ADP and punicalagin were purchased from Sigma-Aldrich (St. Louis, MO, United States), vorapaxar was purchased from Axon Medchem (Atlantic Road, Bristol, United Kingdom), BMS-986120 was purchased from Cayman Chemical (Ann Arbor, MI, United States) and Calcein-AM was purchased from Invitrogen (Carlsbad, CA, United States). PAR1-AP (TFLLRN-NH2), PAR2-AP (SLIGRL-NH2) and PAR4-AP (AYPGKF-NH2) were synthesized from Cosmogenetech Co., Ltd. (Seoul, Korea).

### Cell culture and cell lines

HT29 (human colorectal adenocarcinoma) and MEG-01 (human megakaryoblast) cells were cultured at 37°C and 5% CO_2_. HT29 and MEG-01 cells were grown in RPMI 1640 medium (Welgene Inc., Gyeongsan, Korea). All media were supplemented with 10% FBS, 100 units/mL penicillin and 100 μg/mL streptomycin. HT29 cells were purchased from Korean Cell line Bank (Seoul, Korea) and MEG-01 cells were provided by Joo Hyun Nam (University of Dongguk, Gyeonggi-do, Korea).

### Molecular cloning of plasmid constructs

The PAR1-EGFP Construct was created by inserting the EGFP coding sequence into the pLVX-EIP vector. The method for constructing the vector has been previously described (Heo et al., 2022b). The PAR1 coding sequence was amplified by polymerase chain reaction (PCR) using PAR1 Plasmid (Genbank Accession No. NM_001992) as a template. The clone was provided from Korea Human Gene Bank, Medical Genomics Research center, KRIBB, Korea. The PAR1 primer was designed by replacing the STOP codon with Glycine. (Forward primer: TTT​TTG​AAT​TCA​CCA​TGG​GGC​CGC​GGC Reverse primer: TGGCGTCTAGATCCTCC AGT​TAA​CAG​CTT​TTT​GTA​TAT​G).

### Generation of stable cell line

The PAR1-EGFP cell line was produced by lentiviral transduction. The lentivirus production method has been previously described (Heo et al., 2022b). The produced viral supernatant and culture media were mixed in a ratio of 1:2 and applied to HT29 cells. Successfully transduced cells were selected at concentration of 1 μg/mL of puromycin after 72 h.

### Intracellular calcium measurement

Intracellular calcium levels were measured using a Fluo-4 NW calcium assay kit (Invitrogen, Carlsbad, CA, United States) following the manufacturer’s instructions. Briefly, HT29 cells were plated on 96-well clear bottom black wall plate (Corning Inc., Corning, NY, United States) at a density 2 × 10^4^ cells per well. The wells located in the outermost two rows of the 96-well plate were omitted from analysis since the cell confluence in these peripheral wells differed from the inner wells. After 48 h incubation, each well of the 96-well plates were washed twice in Phosphate buffered solution (PBS, 100 μL/wash) and incubated with 100 μL of assay buffer including Fluo-4. After 40 min of incubation, cells were treated test compounds for 10 min prior to treatment with agonist. The fluorescence was measured using FLUOstar Omega microplate reader (BMG LABTECH, Offenburg, Germany) equipped with custom Fluo-4 excitation/emission filters (485/538 nm). Intracellular calcium in-crease was induced with the application of the indicated agonist with syringe pump. All modulators were dissolved in dimethyl sulfoxide solution (DMSO), and cells were treated with a final concentration of 1% DMSO.

### Immunoblotting

MEG-01 cells were plated on 6-well plates with serum-free medium and incubated overnight. Cells were treated with test compounds and then medium and cells were transferred to E-tubes and centrifuged at 13,000 rpm for 10 min at 4°C. Then, the supernatant was discarded, and the pellet containing cells was lysed with RIPA buffer supplemented with Halt protease and phosphatase inhibitor cocktail (Thermo Scientific, Waltham, MA, United States) and sonication for 2 min. Lysed samples were centrifuged at 13,000 rpm for 20 min at 4°C. Extracted proteins were quantified using Bradford Reagent (Sigma-Aldrich, St. Louis, MO, United States) and 40 µg of total proteins were loaded to each well and separated by 4%–12% Tris-glycine precast gel (Koma Biotech, Seoul, Korea). Proteins were transferred to PVDF membranes (Millipore, Billerica, MA, United States), followed by blocking for 1 h with 5% BSA in TBST (Tris-buffered saline with 0.1% Tween-20). The membranes were incubated with primary antibodies overnight at 4°C with the indicated primary antibodies; anti-p42/44 (Cell Signaling, Cat#9102, RRID: AB_330744), anti-phospho-p42/44 (Cell Signaling, Cat#9101, RRID: AB_331646). Then, the membranes were washed three times in TBST, incubated with horseradish peroxidase-conjugated secondary antibodies for 1 h. After being washed three times, membranes were detected using ECL Plus immunoblotting detection system (GE Healthcare, Piscataway, NJ, United States). All experiments were repeated for three times independently and ImageJ software (NIH, MD, United States) was used for result analysis.

### Molecular docking

The structures of vorapaxar and gestodene were obtained from PubChem (Sayers et al., 2024), while the human PAR1 structure was retrieved from the Protein Data Bank (PDB ID: 3VW7) and used for the simulations (Zhang et al., 2012). AutoDock Vina was employed to perform the docking simulations between the prepared ligands and the receptor. The docking was conducted with grid box dimensions of 40 × 45 × 40 to investigate the binding to the domain. The interaction complexes between the side chains of PAR1 and the compounds were visualized using UCSF Chimera (Pettersen et al., 2004).

### Live cell imaging

HT29 cells stably expressing EGFP-tagged PAR1 were seeded in 96-well clear bottom black wall plate (Corning Inc., Corning, NY, United States) at a density 5 × 10^3^ cells per well. After 24 h incubation, the culture medium was aspirated and exchanged with HEPES buffer solution (140 mM NaCl, 5 mM KCl, 1 mM MgCl_2_, 1 mM CaCl_2_, 10 mM Glucose, 10 mM HEPES, pH 7.4). Live-cell imaging was performed using a Lionheart FX automated microscope (BioTek Instruments, Winooski, VT, United States). Beacons specifying the horizontal and vertical (x/y) offset of the wells were defined using Gen5 software version 3.08 (BioTek Instruments) for each well. Then, Images were then captured through a ×20 objective before and after stimulation. The number of EGFP-PAR1 puncta was automatically quantified using the Gen5 software. Within the Cellular Analysis module, the primary mask and count function was applied, and puncta were counted on the GFP channel, with a threshold intensity value set to 8,000. Object selection parameters were configured to include puncta sized between 0.3–4 μm. The puncta were quantified per cell by dividing the total puncta count by the number of cells analyzed in each experimental group. Between 71 and 90 cells were included in the puncta analysis for each experimental condition.

### Calcein-AM-based assessment of morphological change

MEG-01 cells were gently suspended in HEPES buffer solution, then 1 × 10^4^ cells were dispensed into each well of 96-well glass bottom microplates (Corning Inc., Corning, NY, United States) and allowed to attach to the bottom for 30 min. The cells were incubated with HEPES buffer solution containing calcein-AM (1 μg/mL) with gestodene in the presence or absence of vorapaxar for 30 min. The cells stained with calcein-AM were then treated with PAR1-AP for 30 min and images were captured through a ×10 objective using Lionheart FX automated microscope (BioTek Instruments). Circularity analysis was performed using Gen5 software version 3.08 (BioTek Instruments). Cell circularity was calculated using the primary mask and count function in the cellular analysis module of the Gen5 software. A threshold intensity of 3,000 was set for the calcein-AM fluorescence intensity, and object selection parameters were configured to include objects sized between 15–40 μm. The primary mask was displayed in yellow to facilitate the identification of cellular outlines. Circularity values range from 0 to 1, with one representing a perfect circle and lower values indicating increased irregularity in cell shape. The cell circularity was quantified, and the mean circularity was determined by dividing the sum of individual circularity values by the total number of cells analyzed for each experimental group. The circularity analysis included 307 to 451 cells per each experimental condition.

### Platelet aggregometry

This study was approved by the Institutional Review Board of Yonsei University College of Medicine under approval number 4-2021-0321. Informed consent was obtained from eight healthy volunteers (5 males and 3 females) aged between 23 and 38 years old, who met the study’s inclusion and exclusion criteria. Blood samples were collected from each participant *via* venipuncture into tubes containing 3.8% sodium citrate. The samples were gently mixed by inversion and divided into four cuvettes by 0.5 mL. The blood was diluted with an equal amount of saline and then pre-incubated at 37°C for 15 min with 0.5% DMSO, 30 μM gestodene, 30 μM gestodene combined with 1 μM vorapaxar, or 1 μM vorapaxar. After pre-incubation, platelet aggregation activity was induced by adding 10 μM PAR1-activating peptide to each cuvette and measured using an impedance method in a Whole Blood/Optical Lumi-Aggregometer (Model 700; Chrono-log Corporation, Havertown, PA, United States). Aggregation was recorded for 6 minutes, and the maximum amplitude, slope, lag time, and area under curve were analyzed for the entire recording period. The data were analyzed using AGGRO/LINK®8 software (Chrono-log Corporation, Havertown, PA, United States), and results are presented as mean ± standard deviation.

### Data and statistical analysis

For all statistically analyzed studies, experiments were performed at least three times independently. The experiments were carried out in a randomized manner. The results are presented as the mean ± S.E. Statistical analysis was performed with Student’s t-test (for paired or unpaired samples as appropriate) or one-way analysis of variance (ANOVA) followed by the Tukey’s multiple comparison test for *post hoc* analysis. A value of *p* < 0.05 was considered statistically significant. Concentrations of response curves were fitted in GraphPad Prism 5.0 (GraphPad software, San Diego, United States).

## Results

### Identification of a novel positive allosteric modulator of PAR1, gestodene

**FIGURE 1:**
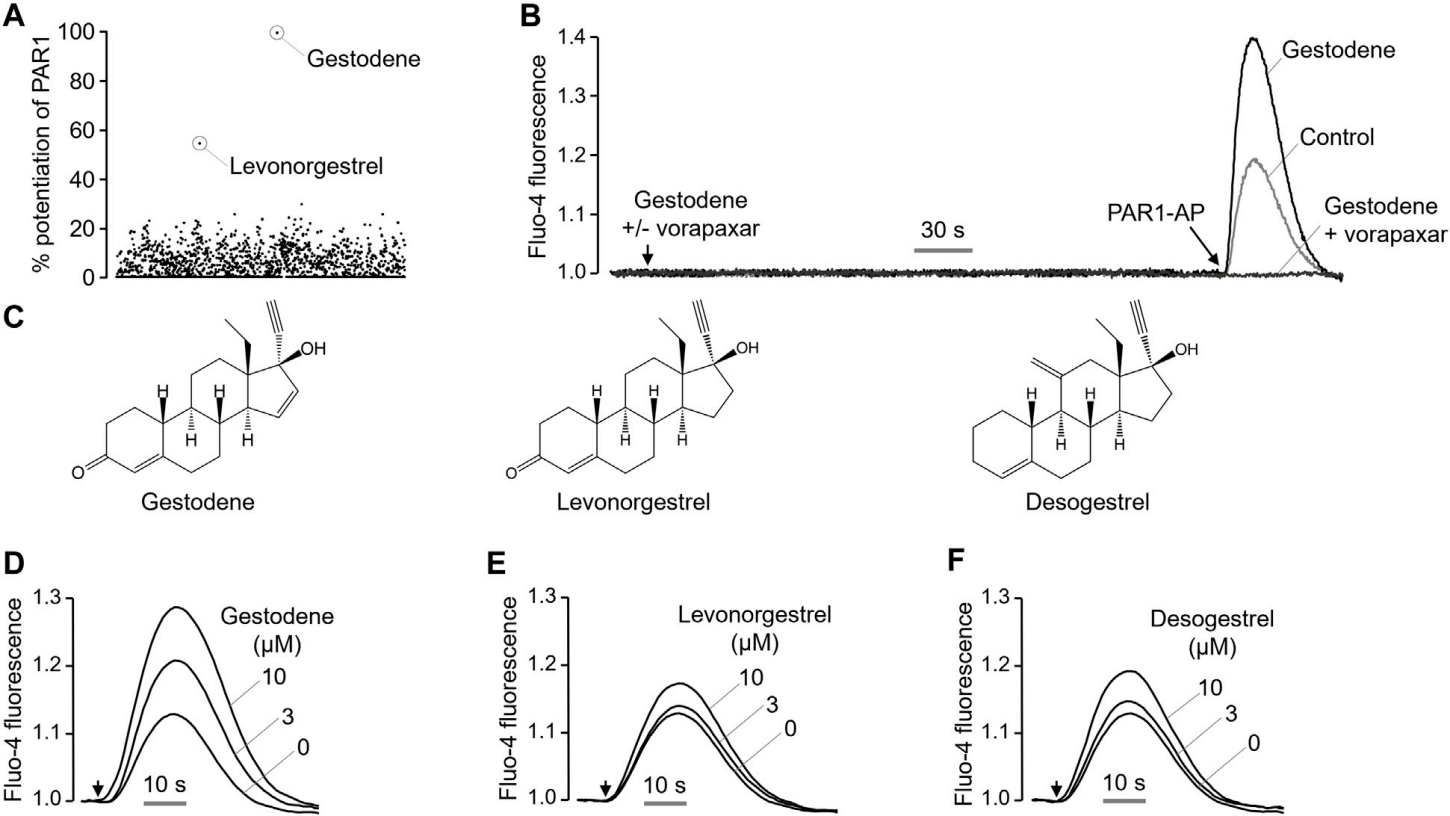
Identification of a novel positive allosteric modulator of PAR1. **(A)** Dot plot shows the effect of 1813 FDA-approved drugs on enhancing PAR1 activity. **(B)** Representative traces of intracellular calcium responses to PAR1-AP (30 μM) in the presence or absence of gestodene (10 μM) in HT29 cells. PAR1 was inhibited with vorapaxar (1 μM). **(C)** Chemical structures of gestodene, levonorgestrel and desogestrel. **(D–F)** Representative traces of intracellular calcium responses to PAR1-AP (20 μM) in HT29 cells pretreated for 10 min with the indicated concentrations of gestodene, levonorgestrel, and desogestrel. Arrows indicate when PAR1-AP was applied.

Excessive activation of PAR1 in platelets by certain drugs may increase the risk of thrombosis. To identify drugs that enhance the PAR1 activity in platelets, a cell based high-throughput screening (HTS) was performed using 1813 chemicals, including approved drugs and investigational drugs. HT29 cells, which endogenously express PAR1, were loaded with the Fluo-4 NW calcium indicator. Cells were treated with 25 μM of the test compounds for 10 min prior to PAR1-AP activation by PAR1-activating peptide (PAR1-AP). Interestingly, the oral contraceptives gestodene and levonorgestrel were found to enhance PAR1 activity (Figure 1A), increasing the PAR1-induced intracellular calcium levels by more than 50%. Gestodene did not by itself induce an increase in PAR1-mediated intracellular calcium levels, but it significantly potentiated the PAR1-AP-induced intracellular calcium levels, and the gestodene-induced PAR1 potentiation was completely blocked by vorapaxar, a potent and selective antagonist of PAR1 (Figure 1B). To determine whether desogestrel, which is structurally similar to gestodene and levonorgestrel, can increase the activity of PAR1, the effects of desogestrel on PAR1 activation by PAR1-AP were observed in HT29 cells. Desogestrel also enhanced PAR1-AP-induced PAR1 activation in a dose-dependent manner (Figures 1C–F).

**FIGURE 2:**
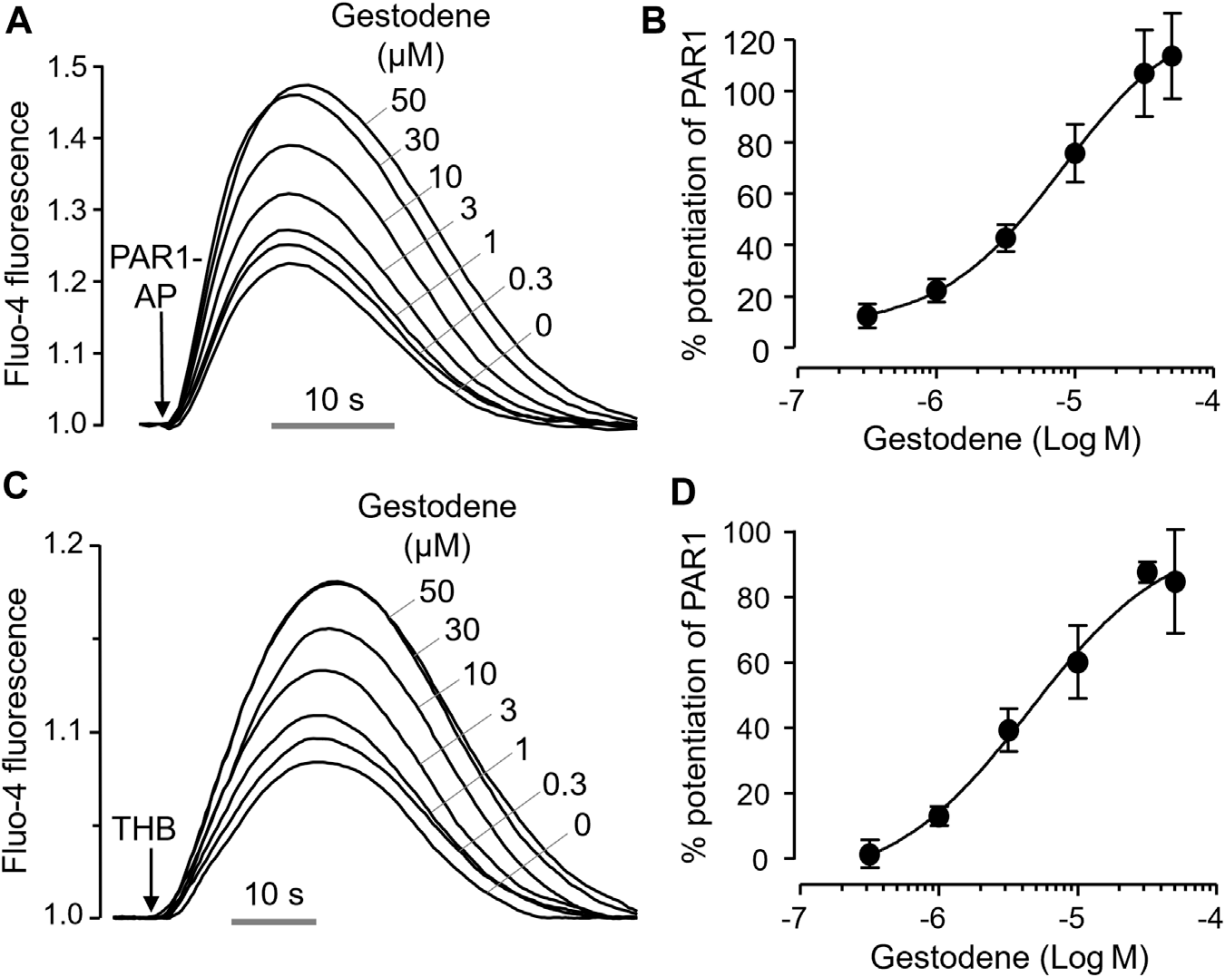
Positive allosteric modulation of PAR1 by gestodene. **(A)** Representative traces of intracellular calcium responses to PAR1-AP (30 μM) in HT29 cells. Cells were treated with the indicated concentrations of gestodene for 10 min prior to PAR1-AP treatment. **(B)** Summary of dose-response (mean ± S.E., n = 6). **(C)** Representative traces of intracellular calcium responses to 3 unit/mL thrombin (THB) in HT29 cells. Cells were treated with the indicated concentrations of gestodene for 10 min prior to thrombin treatment. **(D)** Summary of dose-response (mean ± S.E., n = 3).

To investigate the effect of gestodene on PAR1 activity more precisely, we observed the effect of gestodene on PAR1 activation by PAR1-AP and thrombin, and found that gestodene enhanced PAR1 activation by both PAR1-AP and thrombin with EC_50_ values of 8.15 ± 0.34 μM and 4.54 ± 0.27 μM, respectively (Figure 2).

### Gestodene selectively enhances the activation of PAR1

**FIGURE 3:**
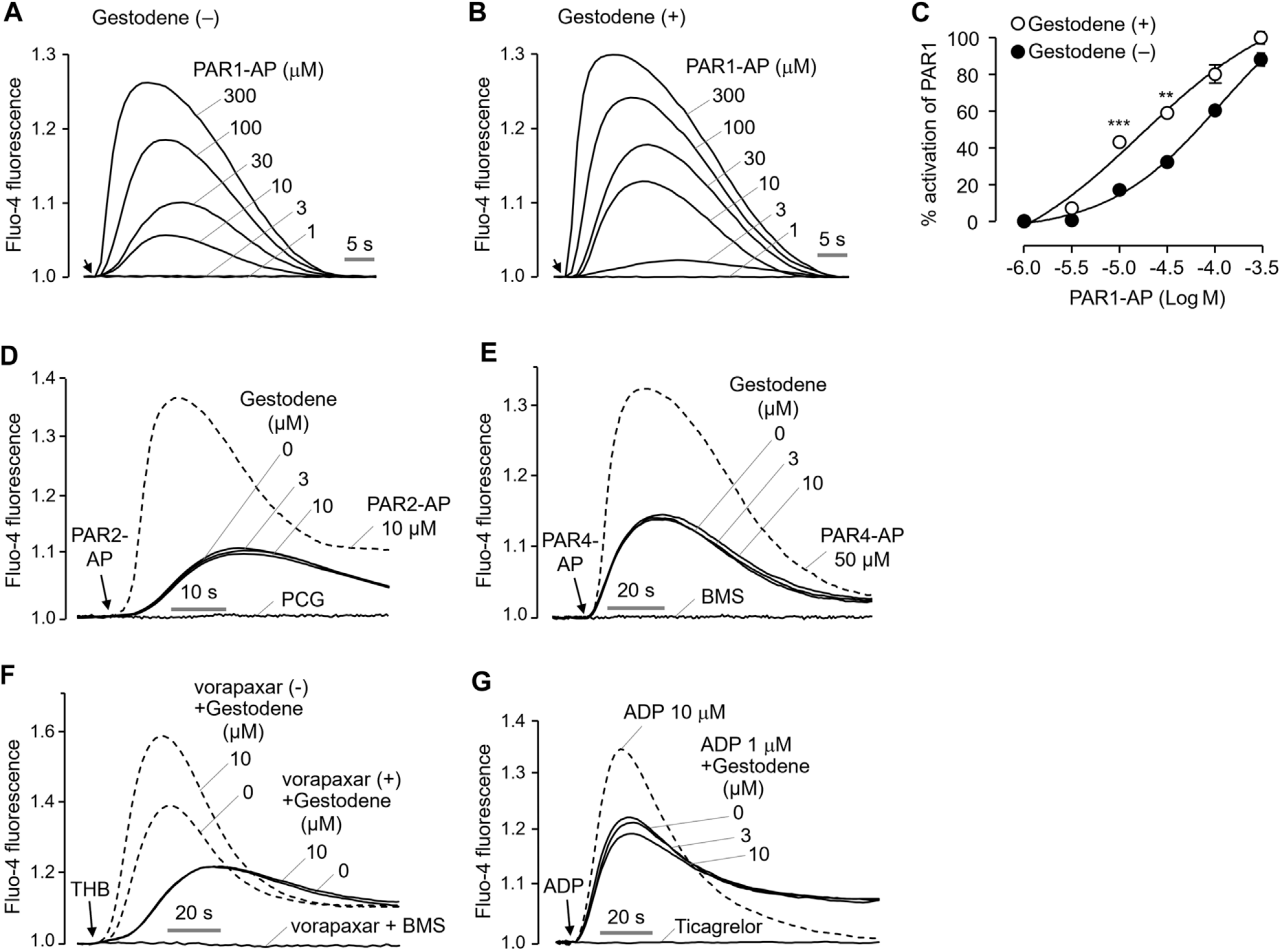
Effect of gestodene on the potency of PAR1-AP and the activity of PAR2 and PAR4. **(A, B)** Representative traces of intracellular calcium responses to PAR1-AP in the presence or absence of gestodene (10 μM) in HT29 cells. Arrows indicate when PAR1-AP was applied. **(C)** Summary of the dose-response (mean ± S.E., *n* = 3). **p < 0.01, ***p < 0.001. **(D, E)** Representative traces of intracellular calcium responses to PAR2-AP and PAR4-AP in HT29 cells. Cells were treated with the indicated concentrations of gestodene for 10 min prior to PAR2-AP (3 μM) or PAR4-AP (30 μM) treatment. PAR2 and PAR4 were inhibited by 30 μM of punicalagin (PCG) and 10 μM of BMS-986120 (BMS), respectively. The dashed line represents full activation of PAR2 and PAR4 by PAR2-AP (10 μM) and PAR4-AP (50 μM), respectively. **(F)** Representative traces of intracellular calcium responses to thrombin in the presence (solid line) or absence (dashed line) of vorapaxar in HT29 cells. Cells were treated with vorapaxar (1 μM) and gestodene (10 μM) for 10 min prior to THB (10 units/mL) stimulation. PAR1 and PAR4 were inhibited by vorapaxar (1 μM) and BMS (1 μM), respectively. **(G)** Representative traces of intracellular calcium responses to ADP in MEG-01 cells. Cells were treated with indicated concentrations of gestodene for 10 min prior to ADP stimulation. The dashed line represents intracellular calcium responses induced by 10 μM of ADP. The intracellular calcium elevation induced by 1 μM of ADP was completely inhibited by 100 μM of ticagrelor.

To investigate the effect of gestodene on the potency of PAR1-AP, activation of PAR1 by PAR1-AP was observed in the presence and absence of 10 μM gestodene. As shown in Figures 3A–C, pretreatment of gestodene significantly enhanced EC_50_ of PAR1-AP by approximately 3.2-fold from 61.17 ± 0.02 μM to 19.13 ± 0.05 μM in HT29 cells. Next, we observed the effects of gestodene on other PARs, PAR2 and PAR4, in HT29 cells functionally express PAR2 and PAR4. As shown in Figures 3D, E, gestodene did not affect the activation of PAR2 and PAR4 by their specific activating peptides up to 10 μM, and PAR2 and PAR4 were completely blocked by their specific antagonists punicalagin and BMS-986120, respectively (Wong et al., 2017; Seo et al., 2020). In addition, to elucidate the effect of gestodene on thrombin-activated PARs, we evaluated the effect of gestodene on thrombin-induced activation of PARs by comparing cells treated with vorapaxar, a selective PAR1 antagonist, to untreated control cells. Upon thrombin stimulation, cells without vorapaxar treatment exhibited potentiation of PAR activity by gestodene. Conversely, in cells pretreated with vorapaxar to inhibit PAR1 activity, the potentiation effect of gestodene on the thrombin-induced increase in intracellular calcium levels was not observed (Figure 3F). Adenosine diphosphate (ADP) is a primary platelet agonist. Therefore, we investigated the effect of gestodene on ADP-induced elevations in intracellular calcium levels in MEG-01 cells. As shown in Figure 3G, gestodene did not significantly alter the increases in intracellular calcium concentrations elicited by a submaximal concentration of ADP.

### Molecular docking analysis

**FIGURE 4:**
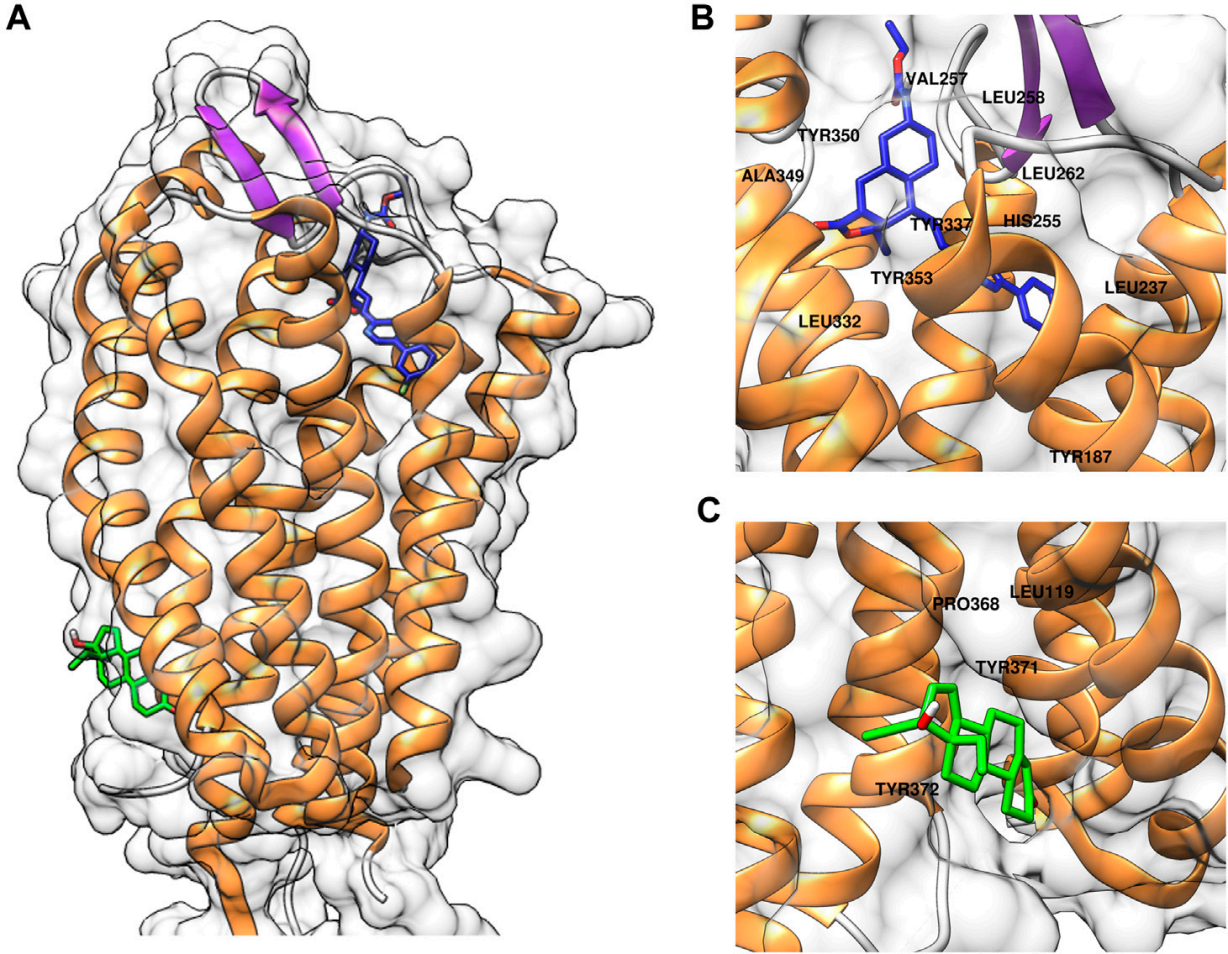
Computational docking simulations of compounds binding to PAR1. **(A)** Predicted binding models of vorapaxar (blue) and gestodene (green) in complex with PAR1. **(B)** Close-up view of vorapaxar (blue) interacting with the orthosteric binding site of PAR1, highlighting key residue interactions. **(C)** Close-up view of gestodene (green) binding to the allosteric site of PAR1, highlighting key residue interactions.

To elucidate the binding mode of gestodene to PAR1, molecular docking simulations were conducted using AutoDock Vina with the crystal structure of PAR1 (PDB ID: 3VW7). Initially, the molecular docking assay was validated using vorapaxar, a known PAR1 inhibitor. The molecular docking analysis revealed that vorapaxar potentially interacts with several key residues of PAR1, including Leu237, His255, Val257, Leu258, Leu262, Leu332, Tyr337, Ala349, Tyr350, and Tyr353 (Figure 4B). These predicted interactions suggest that vorapaxar binds to the orthosteric site of PAR1, which is consistent with previous reports (Zhang et al., 2012; Hidayat et al., 2015). Subsequent molecular docking analysis of gestodene revealed potential interactions with residues Leu199, Pro368, Tyr371, and Tyr372, which are located in the cytosolic domain of PAR1 (Figure 4C). These findings suggest that gestodene binds to an allosteric site, distinct from the orthosteric site where vorapaxar interacts with PAR1.

### Gestodene enhances PAR1-AP-induced internalization of PAR1

**FIGURE 5:**
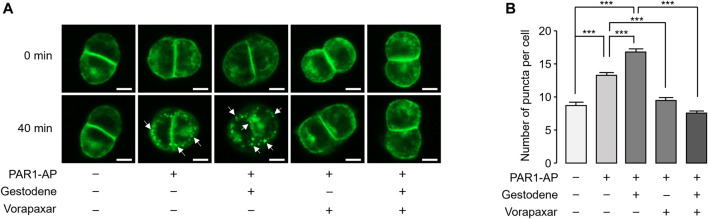
Effect of gestodene on PAR1-AP-induced internalization of PAR1. **(A)** EGFP-tagged PAR1-expressing HT29 cells were pretreated with gestodene (30 μM) in the presence or absence of vorapaxar (1 μM) for 10 min, prior to stimulation with PAR1-AP (30 μM). Changes in cellular localization of EGFP-tagged PAR1 were observed at the indicated time points. White arrows indicate representative internalized EGFP-PAR1 puncta. Images were captured automatically by Lionheart FX microscope. Scale bar = 10 μm. **(B)** Summary of the number of puncta per cell. The number of EGFP-PAR1 puncta were quantified using Gen5 software (mean ± S.E., from left to right, *n* = 73, *n* = 90, *n* = 89, *n* = 71, and *n* = 79 cells were analyzed). *p < 0.05, ***p < 0.001.

Activation of PAR1 by agonists induces internalization of the PAR1 in many cell types, including platelets. To observe the effect of gestodene on internalization of PAR1, EGFP-tagged PAR1 was stably expressed in HT29 cells. PAR1-AP-induced receptor internalization was examined using an automated fluorescence microscopy. PAR1 internalization induced by PAR1-AP was quantitatively measured by the number of EGFP-PAR1 puncta. Notably, gestodene significantly increased PAR1-AP-induced internalization of PAR1, and vorapaxar pretreatment almost completely blocked PAR1 internalization in PAR1-AP-treated cells as well as in cells treated with both gestodene and PAR1-AP (Figure 5).

### Gestodene enhances PAR1 activation and PAR1-Mediated phosphorylation of ERK1/2 in MEG-01 cells

**FIGURE 6:**
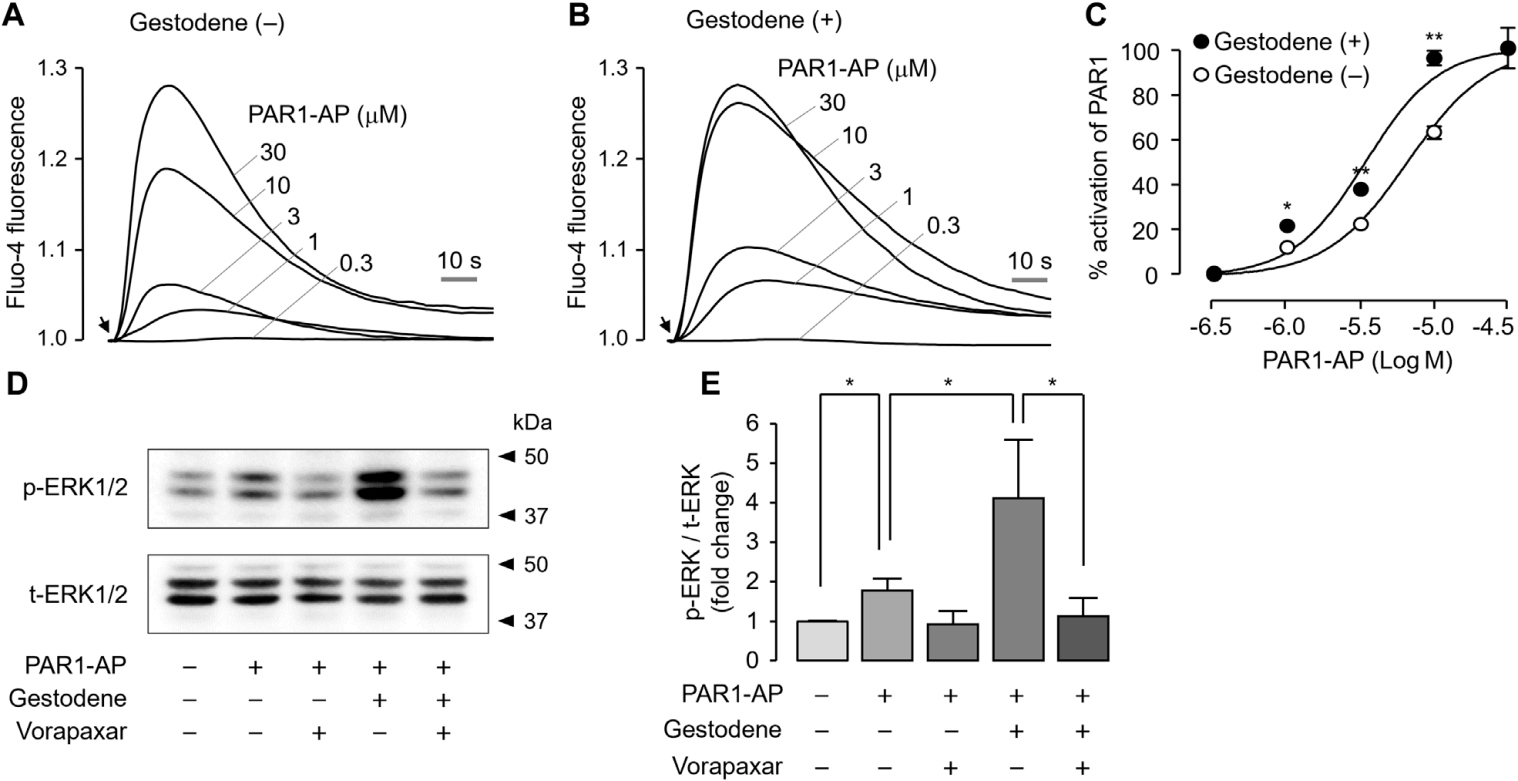
Effect of gestodene on the potency of PAR1-AP and PAR1-mediated phosphorylation of ERK1/2 in MEG-01 cells. **(A, B)** Representative traces of intracellular calcium responses to PAR1-AP in the presence or absence of gestodene (10 μM) in MEG-01 cells. Arrows indicate when PAR1-AP was applied. **(C)** Summary of dose-response (mean ± S.E., *n* = 3). **(D)** Phosphorylation of ERK1/2 was observed after treatment of cells with gestodene (10 μM) for 10 min in the presence or absence of vorapaxar (1 μM) prior to stimulation with PAR1-AP (30 μM) for 5 min **(E)** p-ERK1/2 band intensity was normalized to t-ERK1/2 (mean ± S.E., *n* = 4). *p < 0.05, **p < 0.01.

To investigate the effect of gestodene on PAR1 activity in the human megakaryoblastic leukemia cell line MEG-01 cells, PAR1 was activated by PAR1-AP in the presence or absence of 10 μM gestodene. Gestodene significantly increased PAR1 activation. Pretreatment with gestodene enhanced the EC_50_ of PAR1-AP by approximately 1.9-fold from 6.58 ± 0.04 μM to 3.41 ± 0.04 μM (Figures 6A–C).

Thrombin activates several members of the MAPK family that play important roles in platelet aggregation, including ERK1/2, through PAR1 activation in human platelets (Popović et al., 2012). To investigate the effect of gestodene on PAR1-mediated activation of ERK1/2, phosphorylation of ERK1/2 was studied in MEG-01 cells. As shown in Figures 6D, E, PAR1-AP significantly increased the phosphorylation of ERK1/2, and vorapaxar completely blocked the PAR1-AP-induced phosphorylation of ERK1/2. As expected, gestodene significantly increased PAR1-AP-induced phosphorylation of ERK1/2, and the enhanced ERK1/2 phosphorylation was also almost completely blocked by vorapaxar.

### Gestodene enhances PAR1-Mediated morphological changes in MEG-01 cells

**FIGURE 7:**
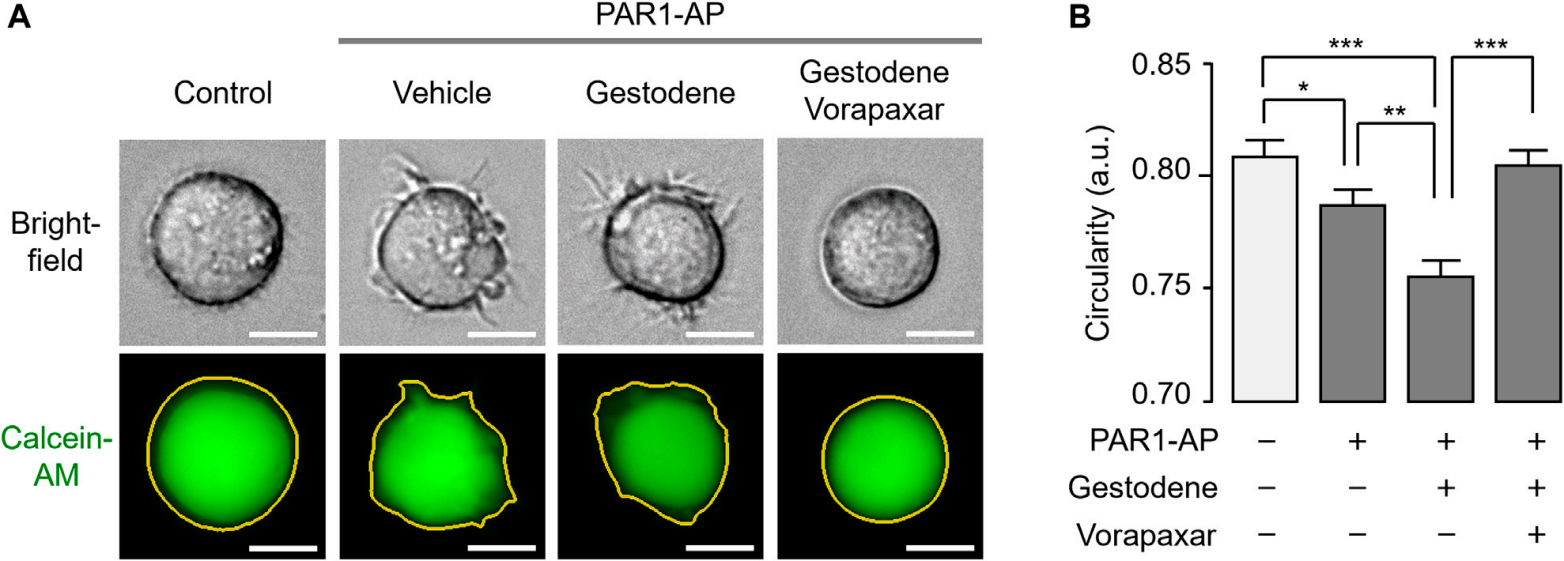
Effect of gestodene on PAR1-mediated morphological changes in MEG-01 cells. **(A)** Representative images of MEG-01 cells used for analysis of circularity measurements. Cells were stained with calcein-AM (1 μg/mL) and pretreated with gestodene (30 μM) in the presence or absence of vorapaxar (1 μM) for 30 min, and then PAR1-AP (30 μM) were applied for 30 min. Yellow lines indicate cell boundaries used to quantify circularity from calcein-AM fluorescence images. Images were captured automatically by Lionheart FX microscope. Scale bar = 10 μm. **(B)** Summary of circularity. Circularity was quantified using Gen5 software (mean ± S.E., from left to right, n = 307, n = 410, n = 451, and n = 417 cells were analyzed). *p < 0.05, **p < 0.01, ***p < 0.001.

In a previous study, we have established a live cell image-based analysis method for the quantification of morphological changes of MEG-01 cells upon thrombin receptor activation (Heo et al., 2022a). To investigate the effect of gestodene on the PAR1-mediated morphological changes of MEG-01 cells, the cells were stained with calcein-AM and the stained cells were imaged 30 min after PAR1 activation. Gestodene significantly potentiated the reduction in circularity of MEG-01 cells induced by PAR1-AP, and the effect of gestodene was almost completely blocked by vorapaxar (Figure 7).

### Gestodene enhances PAR1-Mediated human platelet aggregation

**FIGURE 8:**
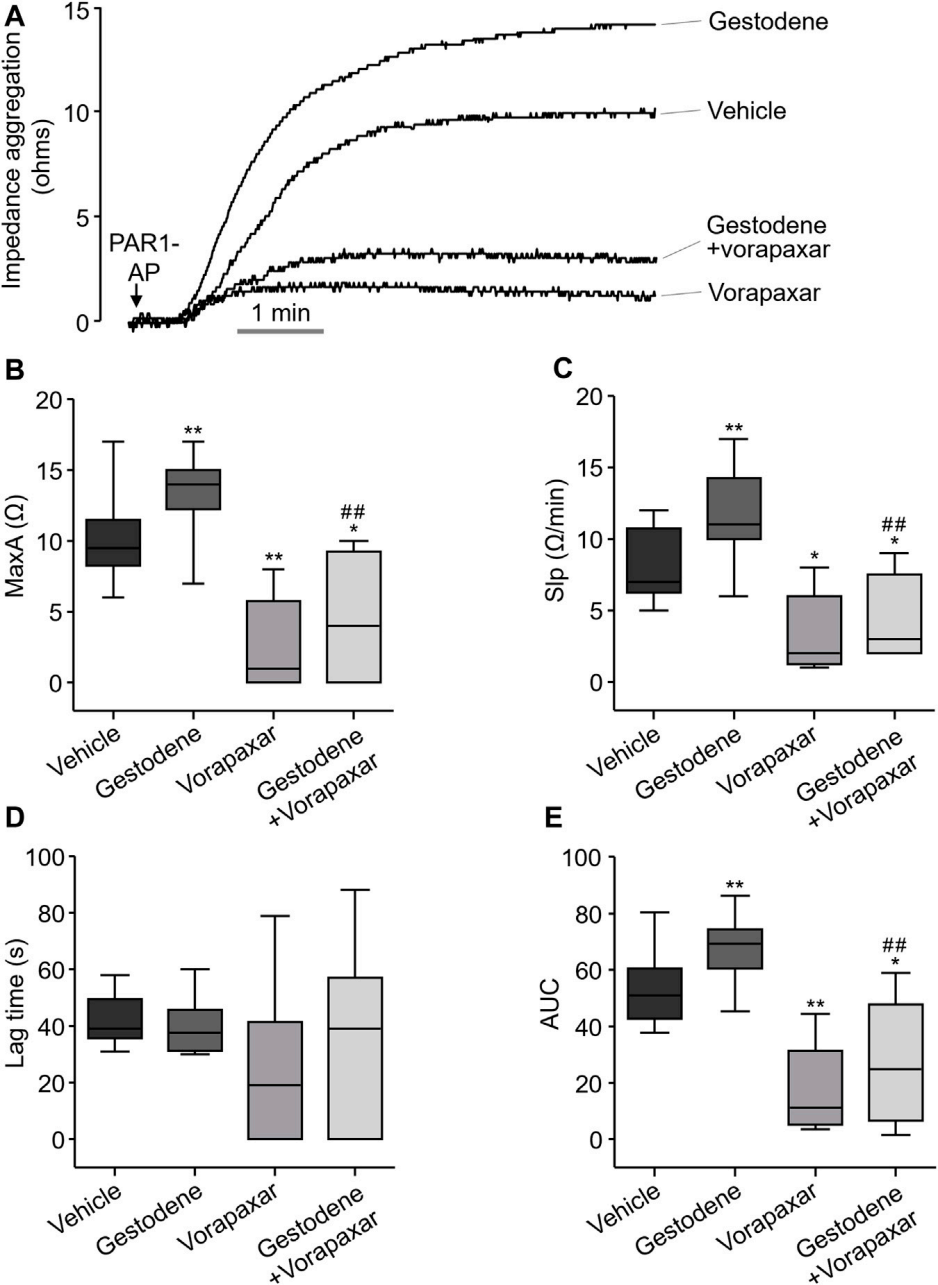
Effect of gestodene on PAR1-AP-induced human platelet aggregation. **(A)** Representative traces of whole blood impedance aggregometry. **(B)** PAR1-AP-induced platelet aggregation expressed as the maximum aggregation (MaxA). **(C)** The slope of platelet aggregation (Slp). **(D)** The Lag time. **(E)** The area under the curve (AUC). Boxplots display median (line), upper and lower quartile (boxes), and maximum and minimum values (whiskers). Gestodene (30 μM) and vorapaxar (1 μM) were pretreated for 15 min before PAR1-AP (10 μM) application (n = 8). *p < 0.05, **p < 0.01 vs. vehicle; ##*p* < 0.01 vs. gestodene group.

Gestodene may increase the risk of venous thromboembolic complications (Spitzer et al., 1996). To investigate whether gestodene can enhance PAR1-mediated human platelet aggregation, the effect of gestodene on human platelet aggregation was observed. Human blood was diluted with an equal volume of saline and then pretreated with gestodene prior to PAR1-AP application. As shown in Figure 8, gestodene significantly increased PAR1-AP-induced maximum aggregation, slope, and area under curve, excluding lag time, compared to vehicle. Notably, vorapaxar significantly decreased the ges-todene-induced increases in maximum aggregation, slope, and area under curve.

## Discussion

PAR1, a thrombin receptor expressed on human platelets, plays a pivotal role in thrombin-induced platelet activation (Angiolillo et al., 2010). The clinical use of a PAR1 antagonist, vorapaxar, has been associated with an increased risk of bleeding due to reduction in platelet activity (Abdulsattar et al., 2011). This finding suggests that excessive platelet activation by PAM of PAR1 may be associated with thrombosis. In the present study, we performed a cell-based HTS to identify known drugs that act as a PAM of PAR1. Subsequently, we investigated whether the agents that can increase PAR1 activity might increase the risk of thrombosis by enhancing PAR1-induced platelet activation. Interestingly, we found gestodene, a novel PAM of PAR1, shows a potent and selective enhancement of PAR1 activity (Figures 2, 3). Gestodene is a synthetic progestogen and third-generation OC.

Interestingly, Lidegaard et al. showed that OCs containing desogestrel and gestodene may double the risk of thromboembolism compared with OCs containing levonorgestrel (Lidegaard et al., 2011). In this study, we demonstrated that levonorgestrel and desogestrel, which are structurally very similar to gestodene, also have the ability to enhance PAR1 activity with different potencies (Figures 1C–F). Notably, gestodene exhibited more potent PAR1-enhancing effect compared to levonorgestrel and desogestrel, and the PAR1-enhancing effect of gestodene was 1.6-fold and 1.5-fold higher than that of levonorgestrel and desogestrel at 10 μM concentration. In addition, Herkert et al. demonstrated that treatment of vascular smooth muscle cells isolated from rat aorta with various progestins (3-keto-desogestrel, progestin, medroxyprogesterone acetate, and gestodene) led to upregulation of PAR1 expression, concomitantly inducing a significant potentiation of procoagulant activity within the vasculature (Herkert et al., 2001). These results suggest that the increased risk of venous thromboembolism associated with gestodene may be partially attributable to an enhancement of both PAR1 expression levels and PAR1 activity. Notably, however, the effects of combined oral contraceptives on platelet aggregation are complex and require careful consideration. Norris et al. demonstrated that the combination of gestodene and ethinylestradiol influence aggregation patterns depending on the type of platelet activator (Norris et al., 1996). While our study focuses on the effects of progestins alone, further research is needed to fully elucidate the interactions between progestins and estrogens in the context of platelet activation and thrombosis risk.

In this study, HT29 cells were employed due to their suitability for investigating the properties of gestodene as a PAR1 PAM. Unlike MEG-01 cells, HT29 cells are adherent and exhibit robust expression of functional PAR1, PAR2, and PAR4. Therefore, HT29 cells were used to assess the PAR1 selectivity of gestodene in comparison to PAR2 and PAR4, under identical conditions (Figure 3). As shown in Figures 1–3, 6, activation of PAR1, PAR2, or PAR4 in HT29 or MEG-01 cells elicited a transient elevation in intracellular calcium levels. This phenomenon is consistent with the rapid desensitization typically observed for most GPCRs upon activation by agonists. In particular, PAR1 shows a more transient calcium signal compared to PAR4 (Shapiro et al., 2000; Chen et al., 2013).

In human platelets, thrombin activated several members of the MAPK family, including ERK1/2, p38, and JNK1, and ERK2 plays an important role in platelet aggregation (Toth-Zsamboki et al., 2003; Yacoub et al., 2006; Mazharian et al., 2007). As shown in Figures 6D, E, gestodene significantly increased phosphorylation of ERK1/2 through PAR1-dependent manner in MEG-01 cells. MEG-01 is a human megakaryoblastic cell line capable of differentiating into megakaryocytes and producing platelet-like particles and is widely used to in the study of *in vitro* platelet production (Ogura et al., 1988; Takeuchi et al., 1998; Isakari et al., 2009). Although this study did not elucidate the effect of gestodene on the activity of MAPK family in human platelet, gestodene enhanced ERK1/2 signaling through PAR1 enhancement in the MEG-01 cell model. This suggests that gestodene may also enhance the activity of MAPK in human platelets as well. Platelets generally have a discoid shape in resting state and then change to a spherical shape when activated, and this change in platelet shape is considered as a prerequisite for platelet aggregation (Hartwig, 1992; Six et al., 2020; Bender and Palankar, 2021). Here, we showed that gestodene significantly reduced the cell circularity of MEG-01 cells (Figure 7). Thus, these results indicate that gestodene may enhance platelet activity and shape change, thereby increasing the risk of thrombosis.

In human coagulation cascade, PAR1-mediated platelet activation plays a critical role (Coughlin, 2005; Rezaie, 2014). Vorapaxar is used in antiplatelet therapy by inhibiting the function of platelets (Hawes et al., 2015; Gryka et al., 2017). The occurrence of moderate to severe bleeding as an adverse effect of vorapaxar serves as clinical evidence highlighting the important role of PAR1 in the process of thrombus formation (Franchi et al., 2015). In this study, we investigated the role of gestodene in human platelet aggregation by PAR1 activation. As shown in Figure 8, platelet aggregation was observed within 6 min following PAR1-AP stimulation, consistent with previous studies (Holinstat et al., 2006). In addition, we found that gestodene enhanced platelet activation induced by PAR1-AP. Notably, we observed a 1.32-fold increase in maximum aggregation (MaxA), a 1.42-fold acceleration in the rate of progression (Slp), and a 1.28-fold augmentation in the overall amount of aggregation (AUC). On the other hand, we did not detect significant alterations in lag time, which represents the initiation time of aggregate formation. Because gestodene does not directly affect platelet activation, but rather exerts its effects after PAR1 activation by PAR1-AP, it is likely that there is a minimal effect on lag time. Interestingly, the aggregation effect was significantly reduced when inhibited by vorapaxar. When gestodene was combined with vorapaxar, the effects of gestodene on MaxA, Slp, and AUC were reduced by 3.05-fold, 2.62-fold, and 2.49-fold, respectively, compared to gestodene administered alone. Taken together, these results suggest that the platelet activation effect induced by gestodene acts through PAR1. Therefore, in terms of clinical perspective, it appears that drugs enhancing the activation of PAR1 may have a sufficient effect on the promotion of thrombosis. To investigate the influence of gestodene on PAR1-AP-induced human platelet aggregation, whole blood was anticoagulated with 3.8% sodium citrate. Considering the calcium chelating effect of sodium citrate reduces extracellular calcium levels and inhibits intracellular calcium signaling mediated by PAR1, a 30 μM concentration of gestodene was employed (Figure 8). However, given that the EC_50_ of gestodene for potentiating thrombin-induced PAR1 activation *in vitro* is 4.54 μM (Figure 2D), gestodene has the potential to augment human platelet PAR1 activity even at plasma concentrations lower than this value. In a pharmacokinetic study of 14 healthy female volunteers, a tri-step formulation cycle increased the C_max_ of gestodene up to 19.4 ng/mL (∼63 nM) (Kuhnz et al., 1993). While circulating gestodene levels are inadequate to markedly augment platelet PAR1 activity systemically, the transient elevated gestodene concentrations in the intestinal following oral administration may be sufficient to transiently and locally potentiate PAR1 activation in platelets, potentially contributing to thrombus formation.

Platelets, red blood cells (RBCs), and leukocytes are intricately involved in the process of thrombosis, wherein PAR1 activation by thrombin plays a pivotal role through the regulation of these cellular components. For instance, activation of PAR1 by thrombin or PAR1-AP in endothelial cells significantly augments the release of von Willebrand factor (VWF) and upregulates the expression of P-selectin, thereby facilitating the adhesion of platelets and leukocytes (Cleator et al., 2006). Furthermore, thrombin elicits the production of platelet-activating factor from endothelial cells, a potent agonist that stimulates the activation of both platelets and leukocytes (Zimmerman et al., 1996). Notably, tissue factor (TF) is the primary initiator of coagulation, and neutrophils contribute to thrombosis *via* the release of TF-bearing neutrophil extracellular traps (NETs), a process critically mediated by thrombin-induced platelet activation *via* PAR1 (Stakos et al., 2015). These findings suggest that the enhancement of PAR1 activity by gestodene in platelets, RBCs, and leukocytes may play an important role in the pathophysiology of venous thrombosis.

In conclusion, our findings demonstrate that gestodene is a *bona fide* PAM of PAR1. Gestodene enhanced the activation of PAR1 induced by both PAR1-AP and thrombin. In addition, gestodene strongly increased PAR1-AP-induced internalization of PAR1, phosphorylation of ERK1/2, and morphological changes in MEG-01 cells. Notably, gestodene significantly enhanced human platelet aggregation. These results suggest that the enhancement of PAR1 signaling by gestodene may be responsible, at least in part, for the increased risk of venous thromboembolism.

## Data Availability

The original contributions presented in the study are included in the article. Further inquiries can be directed to the corresponding authors.
